# Social discounting of pain

**DOI:** 10.1002/jeab.631

**Published:** 2020-10-07

**Authors:** Giles W. Story, Zeb Kurth‐Nelson, Molly Crockett, Ivo Vlaev, Ara Darzi, Raymond J. Dolan

**Affiliations:** ^1^ Max Planck UCL Centre for Computational Psychiatry and Ageing London UK; ^2^ Centre for Health Policy Institute of Global Health Innovation, Imperial College London UK; ^3^ Wellcome Centre for Human Neuroimaging University College London UK; ^4^ DeepMind London UK; ^5^ Department of Psychology Yale University; ^6^ Warwick Business School, The University of Warwick Coventry UK

**Keywords:** social discounting, delay discounting, dread, pain, altruism, kin, humans

## Abstract

*Impatience* can be formalized as a delay discount rate, describing how the subjective value of reward decreases as it is delayed. By analogy, *selfishness* can be formalized as a social discount rate, representing how the subjective value of rewarding another person decreases with increasing social distance. Delay and social discount rates for reward are correlated across individuals. However no previous work has examined whether this relationship also holds for aversive outcomes. Neither has previous work described a functional form for social discounting of pain in humans. This is a pertinent question, since preferences over aversive outcomes formally diverge from those for reward. We addressed this issue in an experiment in which healthy adult participants (*N* = 67) chose the timing and intensity of hypothetical pain for themselves and others. In keeping with previous studies, participants showed a strong preference for immediate over delayed pain. Participants showed greater concern for pain in close others than for their own pain, though this hyperaltruism was steeply discounted with increasing social distance. Impatience for pain and social discounting of pain were weakly correlated across individuals. Our results extend a link between impatience and selfishness to the aversive domain.

Humans and animals tend to be impatient to receive rewards: given a choice people prefer to have rewards sooner rather than later. This implies that reward loses motivational value when delayed, termed *delay discounting* (Frederick et al., 2002; Rachlin et al., 1986). The decrease in value of reward with delay is well described by a hyperbolic function (Kirby & Marakovic, 1995). Similarly, when offered a reward humans exhibit a degree of selfishness: Given a choice people prefer to reward themselves rather than others. This implies that reward for others carries less value than reward for oneself, termed *social discounting*. The value to oneself of rewarding another person decreases with increasing social distance of the other, and this is captured by the same hyperbolic function (Jones & Rachlin, 2006, 2009; Rachlin & Jones, 2008).

Several observers have pointed out an analogy between these two modes of discounting. The problem of how to distribute resources over time can be considered akin to sharing resources between temporally distinct future selves, such that choosing to delay reward entails generosity toward one's future self (Bartels & Rips, 2010; Ersner‐Hershfield et al., 2009; Pronin et al., 2008; Thaler, 1981). Similarly, reciprocal cooperation entails short term sacrifice for the sake of delayed benefit, suggesting that cooperation and future‐oriented behavior are fundamentally linked (Stephens et al., 2002; Stevens & Hauser, 2004). In this sense, delay and social discounting are both discounting of ‘the other’, be it another person, or oneself in the future. As predicted from these theories, social and temporal discounting are indeed correlated across individuals: For reward, impatient people tend to be more selfish (Curry et al., 2008; Rachlin & Jones, 2008). However, this relationship has not hitherto been explored for painful outcomes, where peoples' behavior appears to diverge from the predictions of social and temporal discounting. In this study we explore such relationships in the aversive domain.

Decisions about how to allocate pain across individuals or across time elicit quite different behavior from equivalent decisions for reward. To explain these pain‐specific behaviors, it is necessary to extend conventional models of social and temporal discounting to incorporate additional valuation processes. As we discuss below, existing work has linked these additional processes to subjective or physiological responses to pain. In our ensuing analyses we use terms, such as *dread* and *altruism*, which have previously found application in more subjective or physiological models. We emphasize that their use here does not imply a specific model of an underlying physiological or subjective process, and we instead define each term behaviorally.

## Delay Discounting of Pain

**Figure 1:**
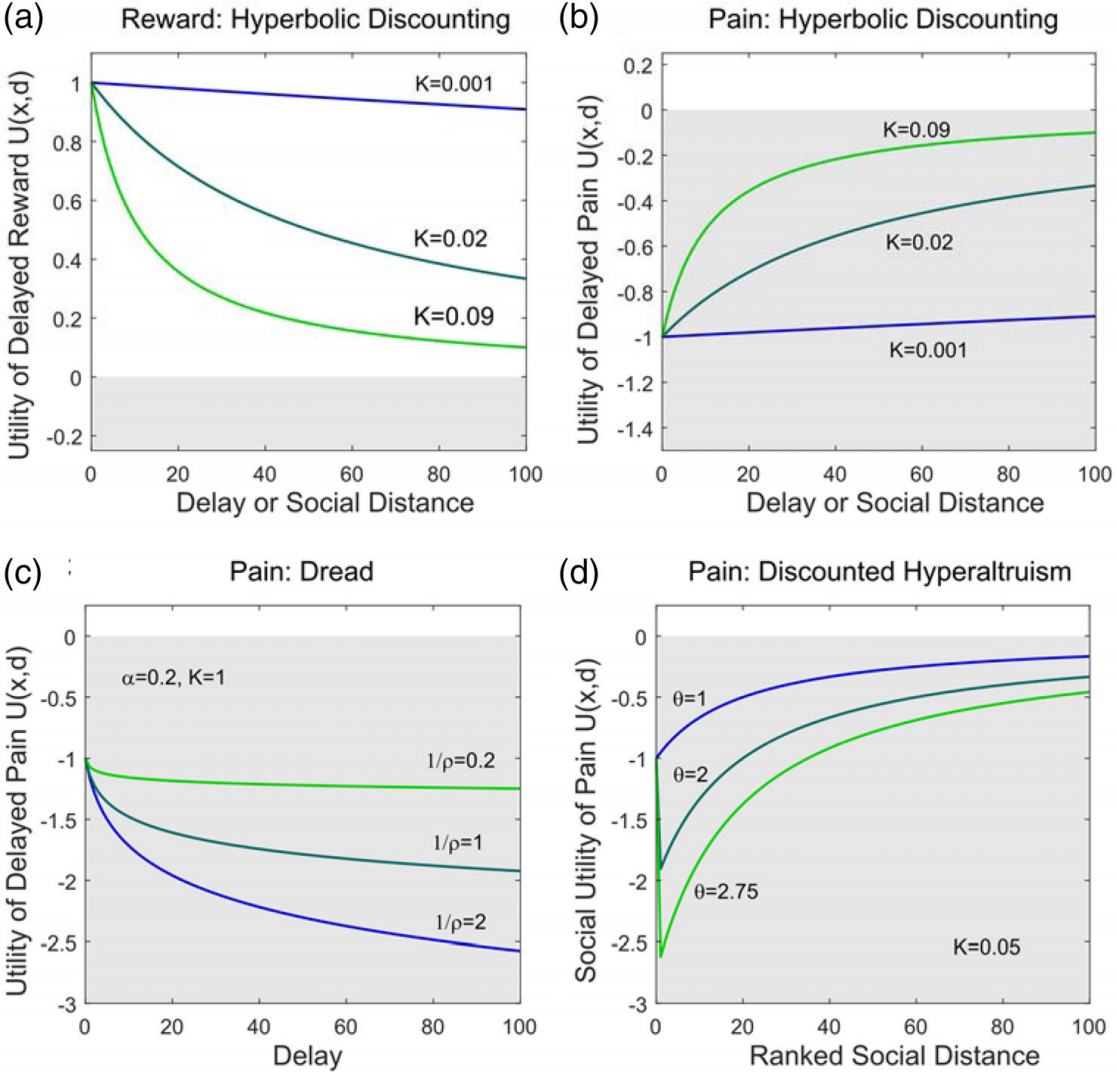
Delay and Social Discount Functions *Note*. Shaded gray areas denote negative utility. **A** Hyperbolic delay discounting of reward of magnitude *x* = 1, with rate K, as shown in Equation 1. Here reward has positive utility, which decreases with delay, motivating choices to speed up reward. **B** Hyperbolic delay discounting of pain of magnitude *x* =  − 1. Here utility of pain becomes less negative with delay, motivating choices to defer pain. **C** Dread of pain as shown in Equation 2: Utility of pain becomes more negative with delay, motivating choices to speed up pain. **D** Social discounting of pain as shown in Equation 4: for *θ* > 1 utility of pain for close others is more negative than the same pain for oneself, motivating choices to take on others' pain. However, this effect is discounted with increasing social distance.

Delay discounting implies that future outcomes carry less motivational weight than immediate ones. In humans and across a range of other species, choices between delayed rewards are well described by a hyperbolic discount function, in which the utility of an outcome of magnitude *x* due to be received after delay *d* is given by:

(1) $$U\left(x,d\right)=u\left(x\right)\left[\frac{1}{1+\mathrm{Kd}}\right]$$

where *u*(*x*) governs the ‘instantaneous’ utility of *x* and *K* is the discount *rate*. Note that an outcome received immediately (*d* = 0) would have value *u*(*x*), while delayed outcomes have lower absolute value, since the overall discount factor, given by the term in brackets, is less than 1 for *K* > 0, as shown in Figure 1a.

As shown in Figure 1b, for a painful outcome, *u*(*x*) would be negative, and increasing delay would bring its disvalue closer to zero. According to discounting, therefore, people ought to defer pain where possible to lessen its disvalue. Strikingly however, most people will opt to hasten an upcoming pain, and many will even endure a more severe pain to speed up its receipt (Badia et al., 1966; Berns et al., 2006; Bertilson & Dengerink, 1975; Cook & Barnes, 1964; Hare, 1966a; Harris, 2012; Loewenstein, 1987; Mischel & Grusec, 1967; Mischel et al. 1969; Story et al., 2013). Contrary to delay discounting, this behavior implies that delayed pain can carry greater disvalue than immediate pain.

Choices to expedite pain are sometimes referred to as implying *negative discounting* (Loewenstein & Prelec, 1991; Van der Pol & Cairns, 2000). Indeed, at first glance, choices to expedite pain might be explained with a negative discount rate (*K* < 0). However, a negative discount rate entails that the disvalue of pain grows with delay at an accelerating rate, such that even a very small pain could become hugely aversive if sufficiently delayed, a prediction that is clearly implausible (see Story et al., 2013). Furthermore, under a hyperbolic model with negative *K*, the disvalue of pain approaches infinity as the denominator approaches zero, producing a discontinuous function.

To address this issue, existing theoretical approaches have considered choice of sooner pain as arising from a process distinct from discounting. Specifically, the phenomenon has been thought of as an attempt to minimize the unpleasant anticipation of future pain, aptly termed *dread*. The notion of anticipatory utility was formalized by Loewenstein (1987), who proposed a model in which decision makers take account not only of the value of an outcome itself, but also the anticipation they are likely to experience while waiting for it. Loewenstein introduced the term dread to refer to “negative utility resulting from contemplation of the future” (p. 667).

In keeping with Loewenstein's notion of dread, an anticipation of pain is itself subjectively aversive (Boucsein & Wendt‐Suhl, 1976; Hodges & Spielberger, 1966; Ploghaus et al., 1999) and engages overlapping physiological and neural responses as pain itself (Boucsein & Wendt‐Suhl, 1976; Grillon et al., 1993; Koyama et al., 1998; Ploghaus et al., 1999). The theory receives further support from the finding that people who exhibit a stronger preference to expedite pain also engage stronger neural responses when passively anticipating pain (Berns et al., 2006).

Dread has also been evoked to explain why monetary losses are discounted at a lower rate than monetary gains (Gonçalves & Silva, 2015; Tanaka et al., 2014; Yates & Watts, 1975). Since people still prefer to defer losses, yet advance the timing of pain, one possibility is that losses incur less dread (Harris, 2012; Loewenstein, 1987). However, the negative effect of monetary losses is also likely to be more temporally extended than the effect of pain, which is usually transient (Harris, 2012; Loewenstein, 1987), and as Loewenstein (1987) demonstrates, for more prolonged experiences discounting tends to dominate effects of dread. A further consideration is that pain, unlike money, cannot be saved or invested, thereby tending to decrease its inherent discounting (Chapman & Elstein, 1995; Harris, 2012).

Here we formalize dread as a cost associated with waiting for pain, that is added to the discounted value of pain itself (Berns et al., 2006; Loewenstein, 1987; Story et al., 2013):

(2) $$U\left(x,d\right)=u\left(x\right)\left[\frac{1}{1+\mathrm{Kd}}+\frac{\;\alpha }{\rho }\log\left(1+\mathrm{\rho d}\right)\right]$$

The first term in brackets represents conventional hyperbolic discounting of pain, while the second term represents the effect of dread. Here dread increases with delay, under an assumption of logarithmic time perception (Han & Takahashi, 2012; Takahashi et al., 2008). (This formulation is also equivalent to the forward‐looking integral of a hyperbolic increase in the expectation of pain across delay, with rate *ρ*, see Supporting Material). The parameter *ρ* governs how dread depends on delay: At lower *ρ* dread becomes more linear in delay, and increases more steeply with delay. α is a scaling parameter which governs the overall contribution of dread. Note that under this model the disvalue of pain increases with delay, albeit at a decreasing rate, as shown in Figure 1c. Under the above framework, choice of sooner pain can be seen to represent a choice to “get pain out of the way”.

In the literature, the term *dread* has been used variously in reference to a subjective and/or physiological state of pain anticipation (e.g., Huang et al., 2017; Richard & Berridge, 2013), to the disutility of this anticipation when choosing the timing of pain or loss (Chapman & Elstein, 1995; Harris, 2012; Loewenstein, 1987; Story et al., 2013; Tanaka et al., 2014), or both (Berns et al., 2006). Here, in keeping with the ethos of this journal, we operationalize dread behaviorally, as a label for the choice of sooner pain, while remaining agnostic to the underlying process. We have taken a similar approach to dread in previous work, which has focused on describing preferences for the timing of pain through behavior analysis (Story et al., 2013). Although the ‘dread’ term in our model does not *necessarily* imply an aversive anticipation of pain, we retain the term ‘dread’ for consistency with previous literature. We note that the model might equally be interpreted in terms of a generic cost associated with waiting for an outcome, and return to this issue in the Discussion.

## Social Discounting of Pain

Social discounting implies that rewarding other people carries less motivational weight than rewarding oneself. By analogy with delay discounting, we can define a discount function across social distance, such that:

(3) $$U_{\mathrm{soc}}\left(x,d\right)=u\left(x\right)\left[\frac{1}{1+K_{\mathrm{soc}}d}\right]$$

Here *U*_*soc*_(*x*, *d*) refers to the social utility to the decision maker of a person at social distance *d* receiving outcome *x*, *u*(*x*) is a utility function over individual outcomes, and *K*_*soc*_ is a *social* discount rate. Note that an outcome allocated to the decision maker themself (*d* = 0) would have value *u*(*x*), while the same outcome allocated to any other person has lower absolute value, since the overall social discount factor, given by the term in brackets, is less than 1 for *K*_*soc*_ > 0.

Social discounting implies that pain for others carries less disvalue than one's own pain, in other words that people will choose pain for others over pain for themselves. By direct analogy with delay discounting, for a painful outcome, *u*(*x*) would be negative, and social discounting brings *U*_*soc*_(*x*, *d*) closer to zero. However, experiments reveal quite the opposite behavior: Participants will endure pain for themselves to prevent pain in anonymous another with whom they will never again interact (Batson et al., 1988; Batson et al., 1983; Davis et al., 2011; Story et al., 2015), and will even pay more money to relieve the pain of another participant than to relieve a similar pain to themselves (Crockett et al., 2014; Crockett et al., 2017; Crockett et al., 2015). Contrary to predictions of social discounting, these behaviors imply that another's pain carries *more* disvalue than one's own. Such behavior has previously been referred to as ‘hyperaltruistic’ (Crockett et al., 2014; 2017; 2015; Story et al., 2015).

These findings resemble preferences for the timing of pain: Both entail enduring pain for one's current self to relieve the pain of another, be it one's future self or another individual. However, while the functional form of temporal preference for pain has been previously described (Berns et al., 2006; Loewenstein, 1987; Story et al., 2013), no previous studies have examined whether and how pain in others is discounted across social distance, and how this might be reconciled with hyperaltruistic behavior. Notably, social discounting as described in Equation 3 cannot produce hyperaltruism.

As considered above for delay discounting, hyperaltruistic choices might be explained with a negative social discount rate (*K*_*soc*_ < 0). However, as we have seen for the case of temporal preferences, this would produce a discontinuous function. Also, a priori the form of social preference for pain likely differs from that of temporal preference. The reason for this is that while the disvalue of pain increases with delay, it is not plausible to suggest by analogy that its disvalue increases with the social distance of the recipient. The latter would imply that people ought to pay more to relieve the pain of a socially distant other, than to relieve the pain of a dear relative or friend, which appears unlikely. Similarly, while dread implies an experience of waiting for pain as the delay elapses, no such concept exists across social distance. Thus, there does not appear to be a direct analogy to dread in the social domain.

In addition, hyperaltruism must have limits to its expression. Findings of hyperaltruism for pain in the laboratory setting appear to overestimate the extent of self‐sacrifice in everyday life (see also Volz et al., 2017); although some noteworthy individuals do endure privation themselves so to relieve the suffering of people previously unknown to them, most people do not spontaneously act in this way. Taken together, a plausible prediction is that people exhibit hyperaltruism with respect to the pain of others with whom they have close ties, but this effect is discounted with increasing social distance. However, although people have been shown to be more willing to suffer pain to themselves in order to relieve the pain of an in‐group member compared to an out‐group member (Hein et al., 2010), this account has been hitherto unexplored.

In keeping with previous models of social discounting for reward, we propose a model for social discounting of pain whereby:

(4) $$U_{\mathrm{soc}}\left(x,d\right)=u\left(x\right)\left[\frac{\theta \;}{1+K_{\mathrm{soc}}d}\;\right]\;\left\{\begin{array}{c} \theta =0\;\text{if}\;d=0\\ \;\theta >0\;\text{otherwise} \end{array}\right.$$

Here *θ* is a parameter that modifies the disvalue of others' pain irrespective of social distance. This parameter can produce hyperaltruism by allowing the overall social discount factor (the term in brackets) to exceed 1. As shown in Figure 1d, values of *θ* > 1 shift the social discounting curve downwards, producing more altruistic behavior towards close others. Higher *K*_*soc*_ steepens the social discount curve. People with high *θ* and low *K*_*soc*_ would be expected to show charitable or caring behavior even towards distant others, for instance victims of war or famine in other countries. By contrast those with high *θ* and high *K*_*soc*_ would be expected to be hyperaltruistic towards close others, but to engage in little altruistic behavior directed outside of their social circle.

Previous work has attempted to delineate processes generating altruistic behavior for pain. For example, an empathy–altruism hypothesis proposes that people relieve others' pain so as to reduce their own discomfort at observing pain in others (Batson et al., 1981, 1988, 1983). This idea accords with findings that most people find observing pain in others subjectively unpleasant (Batson et al., 1988, 1983; Jackson et al., 2005; Lloyd et al., 2004; Milgram, 1965) and that responses to pain in others generate a physiological arousal akin to that provoked by direct pain (Singer et al., 2004). Furthermore, the magnitude of this physiological response predicts a tendency to relieve pain in others (Hein et al., 2011). Other accounts have focused on social norms that prohibit harming others (e.g., Crockett et al., 2014; Fehr & Fischbacher, 2003), suggesting that guilt associated with causing pain in others can produce markedly altruistic behavior. In the current study we remain agnostic to these underlying processes and instead define altruism behaviorally, in terms of social discount parameters.

Our definition accords with previous behavioral approaches to altruism (Fehr & Fischbacher, 2003; Rachlin & Locey, 2011), and is grounded in the notion of revealed preference. In discussing this, Rachlin and Jones (2008) make reference to Simon (1995), who writes:

The conceptual framework employed here obviates the age‐old question about whether an act of giving by one individual to another should properly be labeled “altruism”, or whether instead one is really being “selfish” by making oneself feel good. An individual's discount weights vis‐à‐vis other individuals may be considered a full description of the individual in this connection… (pp. 375‐376).

In keeping with these authors, we consider a social discount function as encapsulating a person's generosity towards another person in a given context, irrespective of underlying motives.

## Current Study

**Figure 2:**
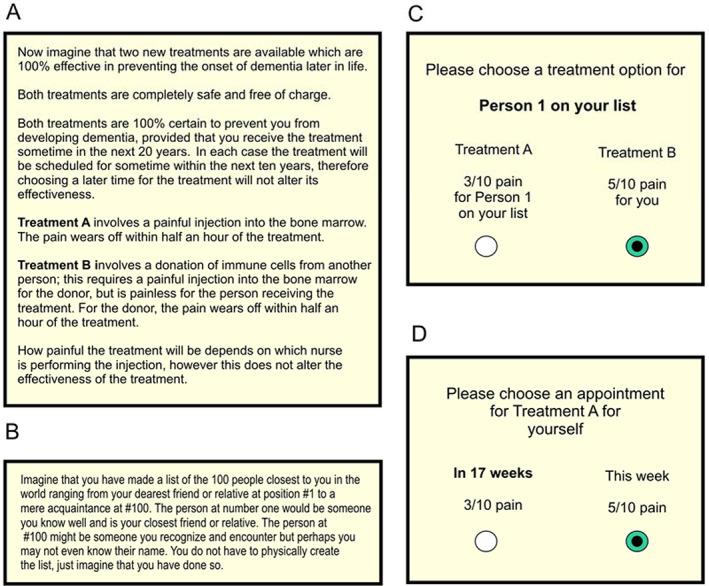
Experimental Protocol for Online Experiments *Note*. **A** Description of alternative painful treatment options for Part 1. **B** Description of social distance manipulation. **C** Example choice from Part 1 designed to test social discounting of pain over social distance. **D** Example choice for Part 2, designed to measure dread.

To explore the functional form of social discounting for pain we conducted an experiment wherein participants chose between two different hypothetical painful medical treatments for another person at a varying social distance from themselves (Fig. 2). Since previous studies have found correlations between delay and social discounting for reward, we also explored for the first time the relationship between delay and social discounting for pain. This is a potentially important area, since models of such preferences and their interactions might suggest novel markers of real‐world caring behavior or dissocial psychopathology, distinct from discounting of reward. To examine this, in a second part of the experiment we also asked participants to choose the timing of the same painful treatments, over delays of up to 8 years either for themselves, or for another person at a specified social distance from themselves.

We use Bayesian model comparison to compare alternative versions of the models described above for their ability to acccount for the observed behavior. Put simply, this statistical approach attempts to find which model is most likely, given the observed data: a quantity termed *model evidence*. Models are scored more highly if they accurately reproduce the observed data, and are penalized for their complexity (e.g., number of parameters). To fit the models we use a hierarchical scheme, namely Bayesian mixed‐effects logistic regression, in which the distribution over model parameters at the group level serves as an empirical prior over individual level parameters. This approach prevents unreliable individual parameter estimates from taking on extreme values.

## Method

### Participants

Healthy participants who had worker accounts on Amazon Mechanical Turk (AMT) were recruited via an advertisement. AMT is an online marketplace for work, now widely used as a method of data collection for psychology experiments (Mason & Suri, 2012). Responses on AMT have been shown to be reliable (Crump et al., 2013) and replicate well‐established findings in the cognitive psychology literature (Rand, 2012). A power calculation indicated that a sample size of 32 participants was required to detect a medium effect (Cohen's *d* = 0.5) at 80% power. Since responses on AMT are likely to be noisier than those obtained in the laboratory, as a heuristic we aimed for double this sample size. We are grateful to a reviewer for noting that an alternative strategy for determining sample size would have been to decrease the expected effect size. Sixty‐seven participants completed the experiment (34 females; mean age 34.0 years, SD = 9.3 years; median yearly income category 25‐30,000 USD; 48/67 university educated). A post hoc power calculation indicated that our study was powered to detect a small to medium effect (*d* = 0.35) at 80% power.

All workers held an AMT ‘Masters Qualification’, meaning that they had consistently demonstrated a high degree of success in performing a wide range of tasks. For each part of the experiment participants were compensated $3, at the current market rate for AMT. Choices were administered via the secure online software, Qualtrics (www.qualtrics.com; Provo, UT).

#### Ethics Statement

All participants gave full informed consent before taking part in the study. The study procedures received approval from the UCL Research Ethics Committee (4418/002). We report all measures, manipulations, and exclusions in these studies.

### Procedure

In Part 1, participants chose between two different hypothetical medical treatments for themselves, which entailed a painful procedure either for themselves, or for a ‘donor’ at a varying social distance from themselves (Fig. 2). The treatments were said to prevent the onset of dementia later in life, to be completely safe, free of charge and 100% effective if received any time in the next 10 years. Participants were told that the painful procedure would involve an injection into the bone marrow causing pain lasting for half an hour. The intensity of the pain was said to depend on the identity of a nurse available to perform the injection, and was described on a 10‐point pain scale. We explicitly told participants that neither the level of pain, nor identity of the nurse, nor the timing of the appointment would alter the effectiveness of the treatment. Treatment A entailed a painful injection into the bone for oneself but no pain for the donor, while Treatment B entailed a painful injection for the donor but no pain for oneself.

As in previous studies, social distance was formalized by asking people to create an imaginary rank ordering of the people closest to them in the world, starting with their dearest friend or relative at position #1 and ending with a mere acquaintance at #100 (Jones & Rachlin, 2006, 2009; Rachlin & Jones, 2008; Fig. 2B). We asked participants to make choices for others at distances of #1, #5, #14, #45 and #97. Choice options followed a symmetrical design, with an equal number of choices in which the pain for self was the more intense and in which the pain for the other was the more intense. Choices were presented in a randomized order. Ninety choices were presented in total, per participant: 18 choices at each social distance. In ‘adjusting self‐pain’ choices the pain intensity for other was fixed at 5/10, while self‐pain intensity varied (from 1/10 to 9/10), whereas in ‘adjusting other pain’ choices the intensity for the other was 5/10 while self‐pain intensity varied (from 1/10 to 9/10). The choice options offered are shown in Table 1. The aim of this procedure is to measure the point at which, as their own pain intensity increases, participants switch from choosing to take on pain themselves to assigning pain to the other person, or *vice versa*. Here, rather than finding indifference points directly, we used model fitting (logistic regression) to find the social discounting parameters which best accounted for each participant's choices.

**Table 1:** Choice Structure

Self/Immediate Pain (Intensity /10)	Other/Delayed Pain (Intensity /10)	Number of Choices at each *d*
*Adjusting Other/Adjusting Delay Choices*		
5	1	2
5	3	2
5	5	1
5	7	2
5	9	2
*Adjusting Self/Adjusting Immediate Choices*		
1	5	2
3	5	2
5	5	1
7	5	2
9	5	2
		Total: **18**

#### Model Fitting Routine

We fitted alternate models of social discounting to the data from Part 1. Each model yielded an estimate of the utility of each choice option, with utilities transformed into probabilities of choosing each option using a softmax function. We used a Bayesian model fitting routine wherein group‐level data is used to generate empirical priors on subject‐level parameter estimates (Huys et al., 2011, 2012). This prevents unreliable parameter estimates at the individual level from taking on extreme values, and yields group‐level estimates of the mean and standard deviation for each parameter, referred to as hyperparameters (see Supporting Material Online for details). The routine used here instantiates a Bayesian mixed effects logistic regression (see Young, 2018). As noted by Young (2018), a Bayesian approach allows the behavior analyst to “quantify the strength of the evidence in favor of one hypothesis versus another hypothesis where neither one has to be the null” (p. 200). Here we make use of this method to evaluate the evidence in favor of alternate models of behavior. The method is particularly useful for comparing large numbers of models, where pairwise hypothesis testing would be cumbersome. Although in the current work we only test a limited number of models, we nevertheless use a Bayesian methodology for better alignment with existing computational approaches (Daw, 2011).

We performed model comparison at the group level using the integrated Bayesian Information Criterion (*BIC*_*int*_). The Bayesian Information Criterion (BIC; Kass & Raftery, 1995) is widely used as a way to score models based on how well they fit the data (likelihood), while penalizing model complexity (number of parameters). It is given by:

(5) $$\mathrm{BIC}=-2\;\log p(C_{1}\ldots C_{N}\left|{\hat{\theta }}^{\mathrm{ML}}\right)+2\left|\theta \right|\;\log\left(\left|C_{1}\ldots C_{N}\right|\right)$$

The first term represents the log likelihood over all choices, *C*, made by *N* subjects at the maximum likelihood parameter estimates, ${\hat{\theta }}^{\mathrm{ML}}$.The second term is a complexity penalty, wherein |*θ*| is the number of free parameters in the model and |*C*_1_…*C*_*N*_| the total number of independent observations. Lower values of BIC indicate a more favorable model fit. (Note that, following convention, *θ* here refers to model parameters in general, rather than the numerator of the social discount equation).

*BIC*_*int*_ is in essence equivalent to conventional BIC, adapted for the purposes of a hierarchical model (Huys et al., 2011, 2012) and is given by:

(6) $$\;{\mathrm{BIC}}_{\mathrm{int}}=-2\;\log p(C_{1}\ldots C_{N}\left|{\hat{\vartheta }}^{\mathrm{ML}}\right)-\frac{1}{2}\left|\vartheta \right|\;\log(|C_{1}\ldots C_{N}|)$$

This differs from conventional BIC in that the first term now refers to the likelihood of the data with respect to the group level *hyperparameters*; calculating this term requires integrating out the individual‐level model parameters (see Supporting Material Online). The number of parameters in the complexity term is accordingly replaced with the number of hyperparameters. As for conventional BIC, lower values of *BIC*_*int*_ indicate higher model evidence, and thereby a better fit to the data. To provide an additional estimate of the goodness of fit of each model we calculated the pseudo‐*R*^2^ using McFadden's formula.

In Part 2, participants made choices between two possible timings for the treatment. In each case, participants were told the timing of the appointment, and how painful the treatment would be on a 10‐point scale. Seventy‐two choices were offered between two possible appointment times for Treatment A either for themselves (termed *self‐now‐self‐later* choices), or on behalf of a friend or acquaintance (*other‐now‐other‐later* choices). We specified that the friend or acquaintance was the 50^th^ person on an imagined list of the 100 people closest to them (termed social distance #50). For each choice, one appointment option was always ‘this week’ (delay 0 weeks), and the other was delayed 4, 17, 52 or 416 weeks.

Ninety choices were presented in total: 18 choices were offered at each delay and presented in a random order. The choice options offered were identical in structure to those used to measure social discounting, as shown in Table 1. In ‘adjusting later pain’ choices the immediate pain intensity was fixed at 5/10, and the delayed pain intensity varied (from 1/10 to 9/10), whereas in ‘adjusting sooner pain’ choices, the delayed intensity was 5/10 and the immediate intensity varied (from 1/10 to 9/10). The aim of this procedure is to measure the subjective cost associated with delay, indicated by the point at which participants switch from preferring the immediate option to preferring the delayed option, or *vice versa*. Here, rather than finding indifference points directly, we used model fitting (logistic regression) to find the dread‐discounting parameters which best accounted for each participant's choices.

## Results

### Data Quality

Response time data for each individual question were not available. However, the median participant took 17 min 57 s to complete the experiment, an average of 4 s per response item. No participants completed the experiment in less than 13 min, that is, all participants spent an average of at least 3 s per item. Three out of 67 participants never chose to allocate pain to the other person. Two out of 67 participants never chose the delayed pain option.

The experiment did not contain ‘catch’ trials. We therefore used modeling analyses to identify participants who appeared to be responding at chance level; to do so for each experiment we compared the best fitting model (see below) with a model generating random responses. Participants for whom the best fitting model did not conclusively outperform a random model (conventional BIC difference < 3) were designated as behaving randomly. For delayed pain choices no participants showed random responding. For social pain choices two participants showed random responding. Since these numbers were low and since our model fitting routine makes use of group‐level data to regularize individual parameter estimates, unless otherwise specified we opted to include all participants in the modelling analyses.

### Hyperaltruism is Discounted across Social Distance

**Figure 3:**
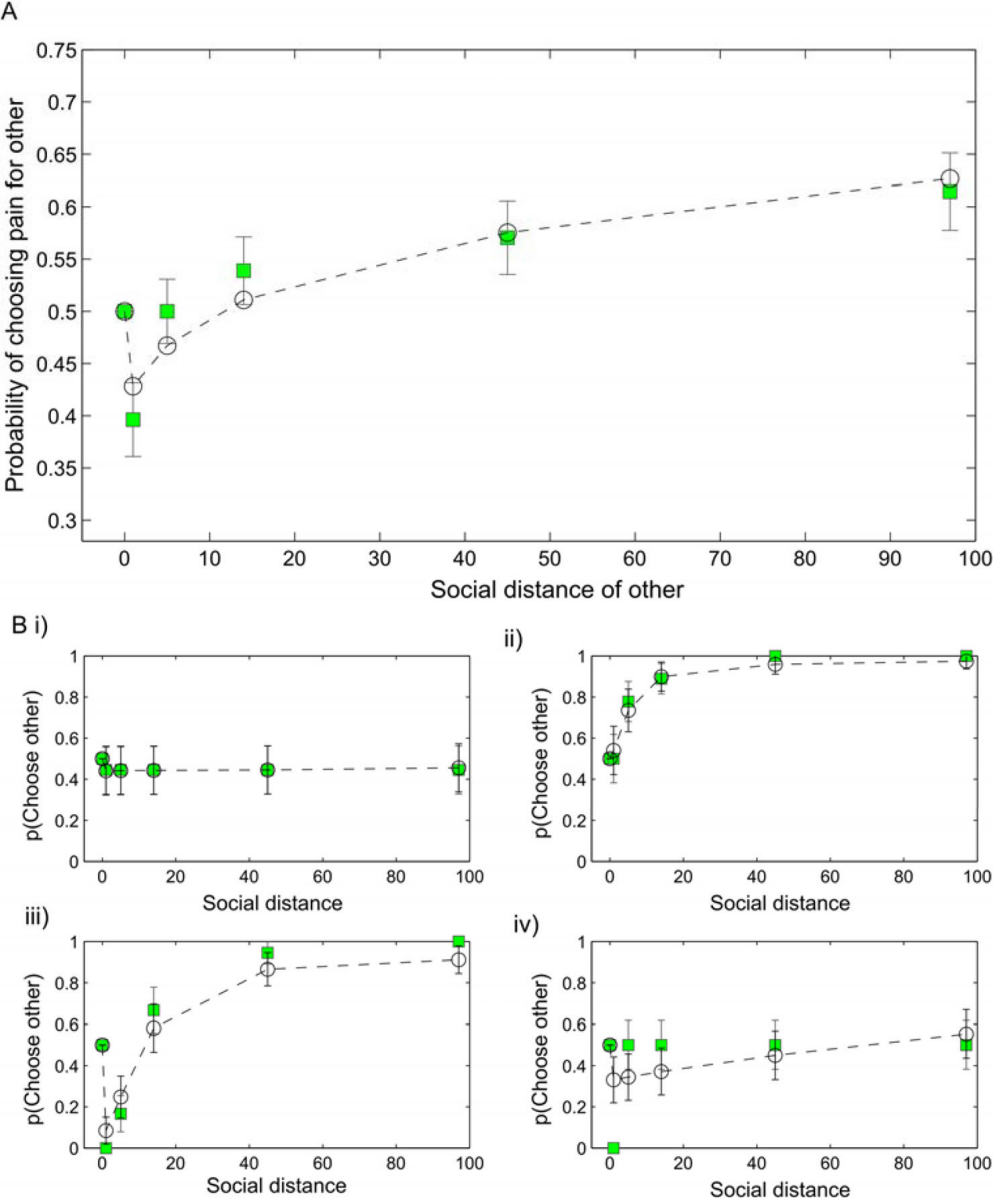
Social Discounting of Pain *Note*. Participants chose between two hypothetical medical treatments for another person: one that entailed pain for the other person and one that entailed pain for themselves. **A** Mean probability of choosing pain for the other person as a function of the other's social distance where #0 = self, #1 = the person's closest friend or relative and #100 = a mere acquaintance. Green squares denote observed data, open black circles joined by dashed lines the maximum a posteriori fit of a Discounted Hyperaltruism model. Error bars show one standard error above and below the mean. **B** Example data from four individual participants. i) Little social discounting of pain, ii) Social discounting of pain, ii) Hyperaltruism for close others discounted with social distance, iv) Hyperaltruism at social distance #1 and no social discounting thereafter. Error bars show one standard error of the binomial distribution.

In Part 1, we arranged the choice structure such that the more severe pain was directed to the self for half the choices, and to the donor for the other half. Thus, a raw metric of relative concern for the donor's pain relative to one's own pain is provided by the proportion of choices on which a participant chooses pain for themselves over pain for the donor. Thus *p*(*choose pain to other*) = 0.5 indicates equivalent concern for self and donor pain, *p*(*choose pain to other*) > 0.5 indicates a greater concern for one's own pain than the donor's pain (i.e., selfishness) and *p*(*choose pain to other*) < 0.5 indicates a greater concern for the donor's pain than one's own pain (i.e., hyperaltruism). As predicted, participants displayed hyperaltruism for close others, that is, chose more severe pain for themselves to relieve a close other of a less severe pain (Fig. 3; mean *p*(*choose pain to other* # 1) = 0.40 95% CI [0.33 – 0.47]), and this tendency was steeply discounted with social distance, such that by social distance #5 participants were indifferent between pain for themselves and others (mean *p*(*choose pain to other* # 5) = 0.50 95% CI [0.44‐0.56]) and exhibited significant social discounting by social distance #45 (mean *p*(*choose pain to other* # 45) = 0.57 95% CI [0.50‐0.64]), that is, treating pain for the person at social distance #45 as less significant than their own.

The model in Equation 4, which we term Discounted Hyperaltruism, provided a good fit to the data (Fig. 3A; mean individual pseudo‐*R*^2^ = 0.71) and conclusively outperformed Simple Hyperbolic Social Discounting (i.e. *θ* = 1; Δ*BIC*_*int*_= 1134), and a Null model (i.e. *θ* = 1, *K*_*soc*_ = 0; Δ*BIC*_*int*_= 2296). Closer examination of the data revealed heterogeneity in subject‐level responses, with some participants showing little social discounting (an example is shown in Fig. 3Bi), some showing a pattern consistent with simple hyperbolic social discounting (Fig. 3Bii) alone (*θ* = 1), and others showing the archetypal pattern (Fig. 3Biii). Some participants appeared to show heuristic responding, showing hyperaltruism for their closest other, and approximate parity between self and other for all other social distances (Fig. 3Biv); this pattern was less well captured by the model.

### Preference for Sooner Pain

**Figure 4:**
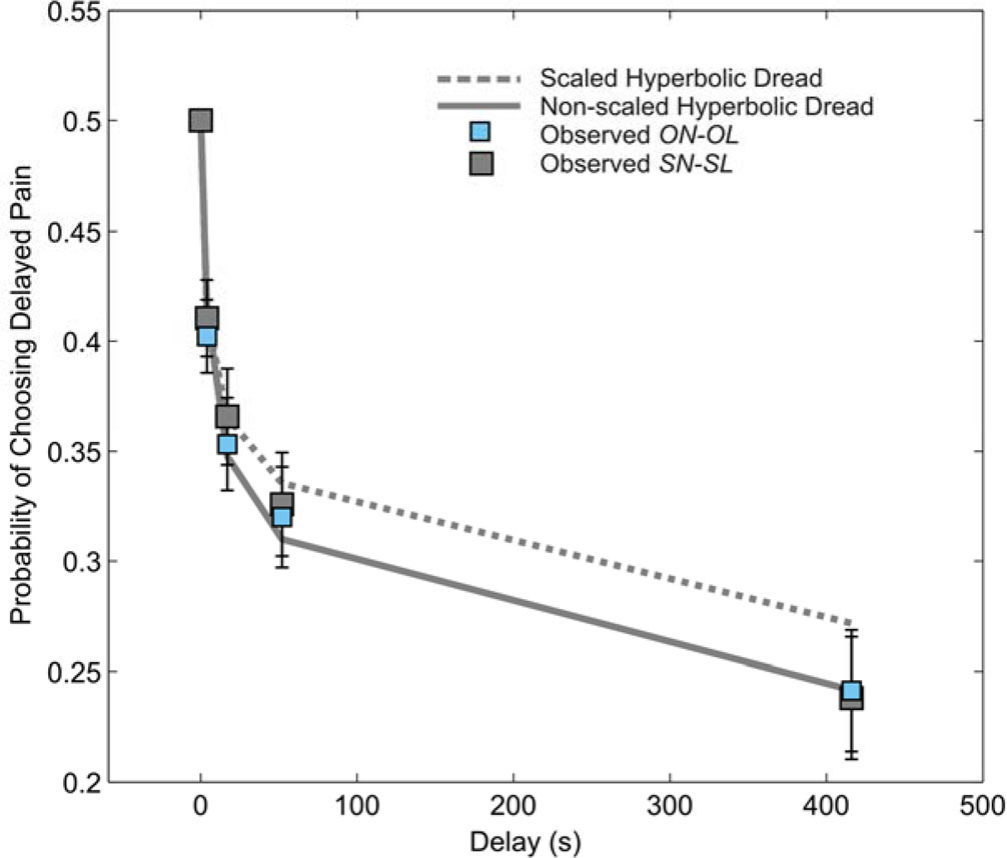
Preference for Sooner Pain for Self and Other *Note*. Mean probability of choosing delayed pain at each delay in *self‐now‐self‐later* (gray squares) and *other‐now‐other‐later* (blue squares) conditions (*N* = 67). Overlaid are maximum a posteriori policies of a Non‐scaled Hyperbolic Dread model (solid gray circles line), and a Scaled Hyperbolic Dread model (dashed gray lines) fitted to *self‐now‐self‐later* choices. Error bars show one standard error above and below the mean.

The proportion of choices at each delay for which participants chose the delayed pain, *p*(*choose later*), provides a raw measure of time preference for pain. Since there was an equal number of options in which the delayed pain was more intense as those where the immediate pain was more intense, *p*(*choose later*) = 0.5 indicates indifference between immediate and delayed shocks, while *p*(*choose later*) < 0.5 indicates a preference for sooner pain, at the expense of increased pain intensity. The curve relating *p*(*choose later*) to delay is shown in Fig. 4, and resembles the decreasing curve observed in previous studies (Story et al., 2015, 2013). It is important to point out here that although this curve resembles the hyperbolic curves seen for reward discounting, it is fundamentally different, since here the relationship implies increasing disvalue of pain with delay (Loewenstein & Prelec, 1991; Van Der Pol & Cairns, 2000). Hyperbolic discounting of pain alone would result in choices to defer pain, that is, *p*(*choose later*) would exceed 0.5 and would increase with delay.

### Fitting Dread‐Discounting Models

In order to derive a parametric estimate of dread, we fitted alternative dread‐discounting models to participants' data from *self‐now‐self‐later* choices (see Supporting Online Material for details of the models). In addition to the exponential models we have tested previously, we tested a model based on hyperbolic discounting, as shown in Equation 2. Based on findings from a related study using a similar design (Story et al., 2020, in submission) we also tested a variant of the above model in which the dread term scales with delay but not with pain intensity:

(7) $$U_{i}\left(x_{i},d\right)=\frac{u\left(x_{i}\right)}{1+\mathrm{Kd}}-\frac{\alpha }{\rho }\log\left(1+\mathrm{\rho d}\right)$$

This unscaled variant showed considerably better correspondence to the data (mean individual pseudo‐R^2^ = 0.92, Δ*BIC* = 487).

To quantify preference for immediate or delayed pain we use numerical integration to calculate an AUC, where this is based on the proportion of choices of delayed pain predicted by the model. Area‐under‐the‐curve (AUC) approaches are commonplace in quantifying discounting for reward (Myerson et al., 2001). Gilroy & Hantula (2018) have shown that calculating the area under the *fitted* discount curve using numerical integration avoids skew associated with linear interpolation between indifference points at longer delays. Unlike discount curves, dread functions are theoretically unbounded in the negative axis; as a result there is no theoretical maximum AUC with which to normalize the resulting integrals. We therefore calculated AUC based on fitted choice proportions, given by the area between the fitted curve relating choice of later pain to delay and a horizontal line at 0.5 for each subject. We normalized this quantity such that AUC = 1 entails always choosing sooner pain, AUC = 0 entails indifference as to the timing of pain, and AUC = ‐1 entails always deferring pain. We observed a group‐level preference for sooner pain, for both self (mean AUC = 0.42, 95% CI [0.33 0.51]) and the other person at social distance #50 (mean AUC = 0.42, 95% CI 0.32 0.51]; Fig. 4). We observed no significant difference in AUC for self and other (*t*(66) = ‐0.12, *p* = .910, Cohen *d* = 0.01).

### Higher Dread Predicts Steeper Social Discounting

To examine the hypothesis that dread is negatively correlated with altruism we plotted social discount curves for subjects grouped into tertiles of dread, measured by the overall weighting on dread, *α*/*ρ*. In this experiment, only 3/67 participants showed a preference to defer pain (defined by AUC > 0). This means that the discount rate *K* is imprecisely defined for the majority of the sample, thereby obscuring interpretability of the dread parameters due to their tendency to trade off against each other. For the purposes of comparing dread with social discounting we therefore excluded these three participants, and fitted the Non‐Scaled Hyperbolic Dread model with *K* = 0.

**Figure 5:**
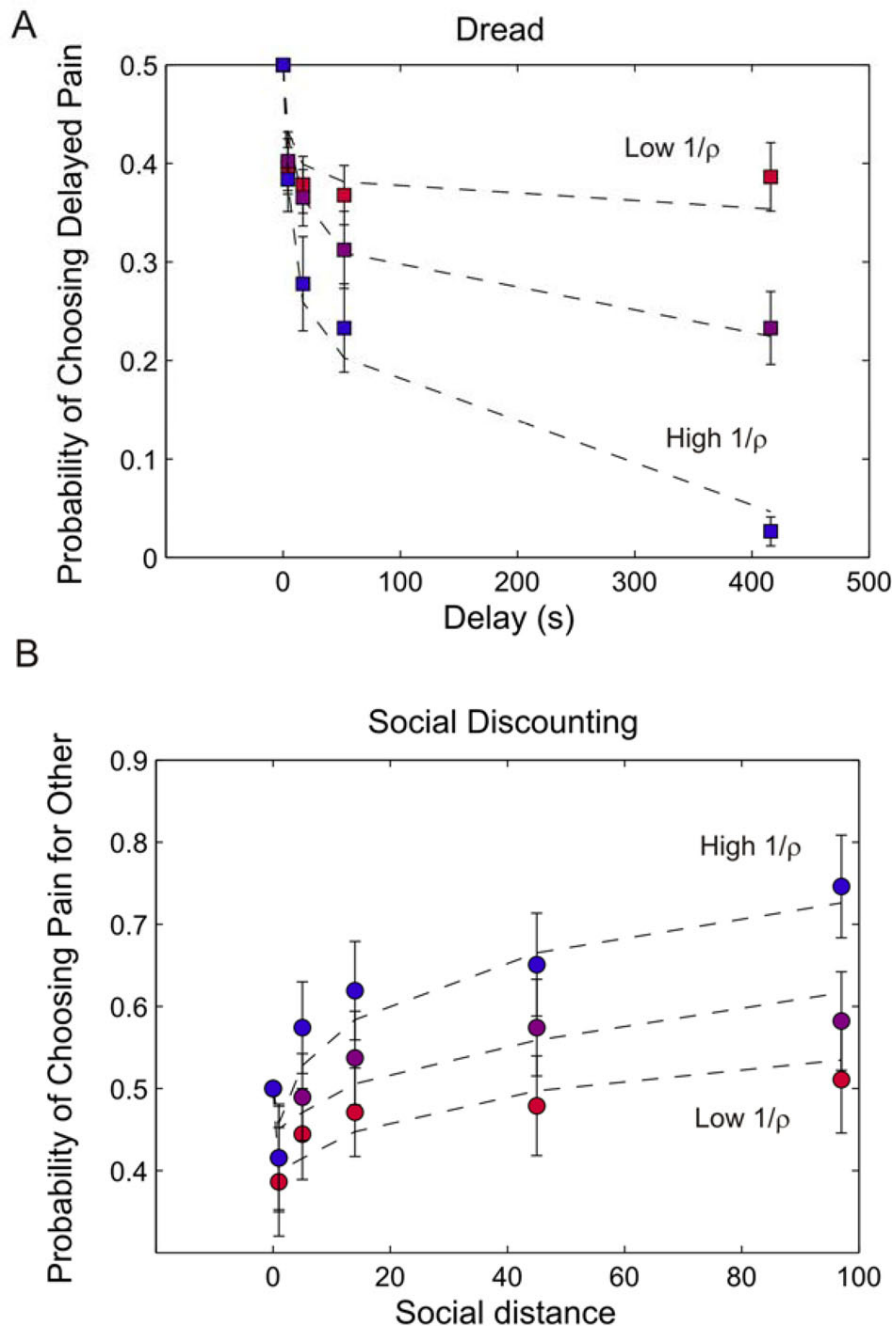
Choice of Sooner Pain Predicts Steeper Social Discounting *Note*. **A:** Mean *p* (choose later) across participants grouped into three tertiles of dread, sorted by the parameter 1/*ρ* . **B:** Mean *p* (choose other) across participants grouped by tertiles of dread, sorted by the parameter 1/*ρ* . Participants in the highest tertile of 1/*ρ* exhibited substantially higher dread, and also showed significantly higher social discounting (*t*(40)= 02.26, *p* = 0.029) than those in the lowest tertile.

Contrary to our prediction, high dreaders showed significantly steeper social discounting (*K*_*soc*_ high dreaders vs. *K*_*soc*_ low dreaders: *t*(40)= 2.03, *p* = .049). To examine which of the two dread parameters was responsible for this effect we sorted the social discount curves by either *α* or 1/*ρ* . Subjects with high *α* exhibited substantially higher dread, but showed no significant difference in social discounting (*t*(40)= 0.17, *p* = .87). By contrast, subjects with higher 1/*ρ* exhibited substantially higher dread, and also showed significantly higher social discounting (*t*(40)= 2.26, *p* = .029; Fig. 5).

We went on to perform regression analyses, with the dread parameters as independent variables; in the first regression model the hyperaltruism parameter *θ* was the dependent variable; in the second model the social discount rate *K*_*soc*_ was the dependent variable. We used weighted least squares where the weightings were given by the precision on the parameter estimates of the dependent variables *K*_*soc*_ and *θ*. Here, subjects for whom these model parameters are more accurately specified contribute more to the fit of the regression line (see Huys et al., 2012). Neither dread parameter emerged as a significant predictor of the hyperaltruism parameter, (*θ* : *β*_*α*_ = 0.09, *p* = .410; *β*_*ρ*_ =  − 0.11, *p* = 0.075). *α* showed no significant relationship with *K*_*soc*_, while *ρ* emerged as a highly significant negative predictor of *K*_*soc*_, consistent with high dread entailing steeper social discounting of pain (*K*_*soc*_ : *β*_*α*_ =  − 0.19, *p* = .11; *β*_*ρ*_ =  − 0.69, *p* < .0001).The same result was obtained when an empirical weighted least‐squares method was used (robust regression, iteratively reweighted least squares with a bisquare weighting function: *K*_*soc*_ : *β*_*α*_ =  − 0.18, *p* = 0.39; *β*_*ρ*_ =  − 0.68, *p* = 0.004).

## Discussion

Here we examined for the first time the relationship between the evaluation of one's own future pain and a sensitivity to pain in others, and whether altruistic responses to another's pain depend on the social distance of the other person. We find support for two novel findings. Firstly, people show greater concern for pain in close others than for their own pain, though this hyperaltruism is steeply discounted (diminishes) with increasing social distance. Secondly, we find a correlation between dread and social discounting, such that people who more strongly prefer immediate pain show steeper social discounting of pain, and thereby tend to be less altruistic overall. In keeping with previous findings, participants chose to speed up the delivery of pain both for themselves or others, even if this entailed an increased intensity of the pain, consistent with an effect of dread (Badia et al., 1966; Berns et al., 2006; Cook & Barnes, 1964; Hare, 1966a; Loewenstein, 1987; Story et al., 2013).

### Social Discounting of Pain versus Money

Social discounting is consistent with evolutionary notions of kin altruism, which proposes that altruism towards related others carries an evolutionary advantage (Curry et al., 2013; Madsen et al., 2007; Schaub, 1996). Our finding of social discounting for pain extends previous findings of hyperaltruism towards close others for money (Rachlin & Jones, 2008), whereby some people prefer to assign a hypothetical monetary reward ($75) to their closest friend or relative (Person #1) than to receive a larger sum themselves (e.g. $80). Rachlin and Jones (2008) note that hyperaltruistic behavior is irrational in the monetary context, since participants could take the $80 for themselves and give it to Person #1. The same authors speculated that, in addition to wishing to signal their closeness to Person #1, people may have chosen the hyper‐generous option due to an implicit cost of having to transfer money, or as a self‐control device to prevent them from keeping the money for themselves. That we find hyperaltruism for close others for painful outcomes, which are nontransferrable, supports a more intrinsic charitable motive, in keeping with kin altruism.

We show support for a model of social discounting in which the net degree of altruistic behavior depends on both the degree of discounting over social distance (*K*_*soc*_) and an additional ‘altruism factor’ (*θ*) that is independent of social distance. Those with a high altruism factor and low social discounting (high *θ*, low *K*_*soc*_) would be expected to show charitable or caring behavior even towards distant others, for instance victims of war or famine in other countries. By contrast, those with a high altruism factor but steep social discounting (high *θ*, high *K*_*soc*_) would be expected to be protective of close kin, but to engage in little altruistic behavior directed outside of their social circle. These categories appear to have high face validity. A future line of investigation might be to compare these parameters for pain with those for money. Existing studies directly comparing generosity for pain and money demonstrate more charitable behavior with painful outcomes (Davis et al., 2011; Story et al., 2015), however to our knowledge no studies have examined this across social distance to test whether the effects are attributable to higher *θ* or lower *K*_*soc*_.

### Applied Social Discounting of Pain

Further research is also required to establish how social discounting of pain relates to real‐world behavior, either charitable or antisocial. Existing work has linked social discounting of money to a range of real‐world behavior. A recent study has demonstrated lower social discounting of reward in extraordinarily altruistic people who have donated a kidney to a stranger (Vekaria et al., 2017), while steeper social discounting has been demonstrated among boys with externalizing (antisocial) behavioral problems (Sharp et al., 2011). Further applied work in this vein might also examine aversive, as well as monetary, outcomes. The current study illustrates that such preferences can be readily elicited using hypothetical painful scenarios.

Other authors have examined the effect of state‐based changes on the social discount curve for reward. Some such models have also examined the effects on the numerator term in the social discount model, namely, *θ*. For example, Wu et al. (2019) showed that testosterone administration in males increased social discounting for distant others, but had no effect on generosity towards close others. Strikingly, Margittai et al. (2015) showed that experimentally induced psychosocial stress appeared to have the reverse effect. Stress increased the numerator term, but had no effect on the social discount factor, manifest as greater generosity towards close, but not distant, others; a follow on study (Margittai et al., 2018) demonstrated that oral administration of hydrocortisone had the same effect. Further work is needed to investigate influences on the numerator term, in particular to disentangle effects of the instantaneous utility term from the effect of *θ*, since these enter multiplicatively into the numerator. Painful stimuli, which allow the form of instantaneous utility to be elicited directly using willingness to pay, offer a route to achieving this.

### Positive Correlation between Dread and Social Discounting of Pain

Previous work suggests that the ability to wait for future rewards and the ability to understand the mental states of others are linked. For instance, temporal discounting for reward and altruistic behavior have been shown to be correlated across individuals (Curry et al., 2008; Rachlin & Jones, 2008), and both are impaired in Borderline Personality Disorder (Bateman & Fonagy, 2004). Along these lines a tendency to expedite pain so as to mitigate dread might be conceptualized as a future‐oriented behavior, akin to showing altruism towards one's future self. Indeed, both dread and altruism for pain have been shown to relate to the strength of physiological response to imagined pain: People who show greater anticipatory brain responses to pain are more likely to expedite pain rather than delay it (Berns et al., 2006), and people who show greater skin conductance responses to pain in others are more likely to choose to relieve another's pain (Hein et al., 2011). In keeping with this idea, people with higher trait psychopathy have been shown to be less likely to choose to expedite their own impending pain (Hare, 1966b) and show diminished physiological responses to the anticipation of pain in others (Caes et al., 2012). By this reasoning dread might be associated with lower social discounting of pain. Strikingly however, and contrary to our prediction, we found evidence that ‘higher dreaders’ showed *steeper* social discounting for pain.

Our data do not permit firm conclusions regarding the reasons for this correlation. However, a possible interpretation is that choice of sooner pain represents more a generic form of impatience than previously thought. We found that preference for sooner pain was best accounted for in terms of waiting cost that scaled with delay, but not with pain intensity. This finding is difficult to reconcile with previous models of dread, which focus on the aversive anticipation of pain, a quantity that would be expected to scale with pain intensity. Imagine for instance that you are contemplating either a trivially painful routine dental check‐up or a considerably more painful dental procedure. Our results suggest that the overall disvalue of the very painful procedure would still be greater than the routine check‐up but that the effect of delay on the disvalue of each would be identical.

The superior fit of a nonscaled model suggests that choices to expedite pain might not solely result from a desire to minimize the anticipation of pain, so much as a desire to reduce a generic cost associated with waiting. Notably reframing *dread* as *impatience* does not require any change to the form of our model, since the model does not specify the processes underlying a tendency to expedite pain. It is possible that a similar impatience term also contributes to discounting of reward (see for example Gonçalves & Silva, 2015). Such a reframing would make the observed correlation between impatience for pain and social discounting congruent with the correlation between delay discounting and social discounting seen for reward. There follows a strong prediction that impatience for pain and for reward ought to be correlated, indicating an important direction for further research.

### Interactions between Dread and Social Discounting of Pain

A further interesting direction for future work concerns how delay and social discounting of pain interact. A pertinent question, for example, is whether effects of dread and social discounting are multiplicative or whether dread is revealed differently when choosing for others. Here we found that a preference for sooner pain was equivalent whether participants chose regarding their own pain, or that of another person at social distance #50. Notably however, in social discounting choices the mean participant showed neither marked social discounting nor hyperaltruism for a person at social distance #50, therefore further exploration is required to establish whether dread interacts with social discounting effects across a range of social distances. We have examined this in an additional study, submitted to this journal, in which we also elicit choices across both domains, for example pain for oneself now, versus for another person in the future, and vice versa (Story et al., 2020).

### Factors in the Valuation of Future Pain

The model described here is challenged to disentangle the effects of discounting and dread within a given individual. We are grateful to a reviewer for the suggestion that measuring temporal preferences for *past* as well as future pain might offer a means to parse the two effects. Prior research has shown temporal discounting of past events to be lawful and also hyperbolic in form (e.g., Yi et al., 2006). Since dread presumably is not contained within events in the past, measuring discounting of past painful events could help to isolate the contribution of dread.

Finally, there are plausible reasons why choices to expedite pain might depend on factors other than dread of pain. Firstly, in many real‐world situations people choose to endure pain or discomfort so as to obtain an associated reward, for example having an immunization to prevent the possibility of illness, doing exercise to improve overall wellbeing, or working to earn a wage. If the rewards accrue at approximately the same time as the pain and outweigh its disvalue, then discounting of the net benefit could motivate speeding‐up the pain–reward combination. Secondly, it is often the case that painful experiences tend to get worse over time, making it rational to face them sooner: For instance in the real world the timing of a dental appointment might be brought forward to relieve worsening dental pain. Although our scenarios attempt to control for these factors, these prior assumptions may nevertheless influence people's experimental choices. Further experimental work is required to disentangle these possibilites.

## Supporting information

**AppendixS1.** Supporting Information.
