# Supplementary material for: Social discounting of pain
Source: J Exp Anal Behav. 2020 Oct 7;114(3):308–25. doi: 10.1002/jeab.631 (PMC8436752; doi:10.1002/jeab.631)
Supplement: Supplementary file 1 — AppendixS1. Supporting Information. [file JEAB-114-308-s001.docx]

Social Discounting of Pain

Giles W. Story, Rony Chowdhury, Zeb Kurth-Nelson^,^ Molly J. Crockett, Ivo Vlaev, Ara Darzi, Raymond J. Dolan

**Supporting Material Online**

**Fitting Alternative Models of Dread-Discounting**

As described in the main text we fitted alternative models of dread to participants choices. We first tested a model of dread proposed by Loewenstein, which we term ‘Exponential Dread’ (Loewenstein, 1987). Under this model, moment-by-moment anticipation is assumed to increase exponentially as pain is approached, and dread is taken to be a prospective integral of this. Here we used Loewenstein’s original formulation, rather than a discrete time version we described previously (Story et al., 2013).

The model is summarized here for completeness, and describes the utility for a decision-maker at time $t=0$ of anticipated pain starting at time $t=d$. Pain itself is assumed to produce a constant stream of disutility beginning at time $d$ and lasting for duration, $L$, where the instantaneous utility of pain is given by:

$$u\left( x \right)=-x-bx^{2}$$

(S1)

Here $b$was set to 0.01, producing very slight convex disutility for pain. The value of pain,$P$, at $t=0$is then given by the exponentially discounted integral of this stream of disutility:

$$P\left( x,d,L \right)= \int_{d}^{d+L} u\left( x \right)e^{-rt} dt$$

(S2)

In addition, whilst waiting for pain, the decision-maker is assumed to derive instantaneous disutility from anticipation, $\left( A \right),$which is given by the (exponentially discounted) expectation of pain at each moment, $t\leq d$:

$$A\left( x,d,t,L \right) =\alpha\int_{d}^{d+L} {u\left( x \right) e}^{-\rho\left( \tau-t \right)}d\tau= \frac{\alpha}{\rho} {u\left( x \right) e}^{-\rho\left( d-t \right)}\left( 1-e^{-\rho L} \right)$$

(S3)

Here $\rho$ is an exponential discount rate governing the rate of increase in momentary anticipation as pain draws nearer, and is distinct from the main discount rate. When evaluating delayed pain from $t=0$, decision-makers are assumed to derive disutility from two sources, the prospective disutility of pain itself, and a prospective estimate of the total moment-by-moment anticipation they can expect to experience whilst waiting for pain.

Under Loewenstein’s formulation both sources of utility are simply added together where each is assumed to be exponentially discounted with rate, $r$, giving:

$$U\left( x,d,L \right) =P\left( x,d,L \right)+D\left( x,d,L \right)$$

(S4)

The second term, referred to as dread, is given by:

$$D\left( x,d,L \right)=\int_{0}^{d} A(x,d,t,L)e^{-rt}dt$$

(S5)

Giving:

$$U\left( x,d,L \right) = \frac{\alpha}{\rho}\int_{0}^{d} u\left( x \right)e^{-\rho\left( d-t \right)}\left( 1-e^{-\rho L} \right)e^{-rt}dt + \int_{d}^{d+L} u\left( x \right)e^{-rt} dt$$

(S6)

The first integral denotes prospective utility from dread, the second prospective utility from pain itself. Integrating both terms gives:

$$U\left( x,d,L \right)=u\left( x \right)\left[ \frac{\alpha}{\rho\left( \rho-r \right)} \left( e^{-rd}- e^{-\rho d} \right) \left( 1-e^{-\rho L} \right)+ \frac{1}{r}e^{-rd}\left( 1-e^{-rL} \right) \right]$$

(S7)

The contribution of dread is governed by the parameters $\alpha$ and $\rho$. $\alpha$ can be viewed as a regression weight on dread, representing an individual’s propensity to dread future pain, while $\rho$ determines the change in dread as pain is delayed further into the future. In total the model has four free parameters at the subject level: $\alpha$,$\rho$, $r$, and the softmax inverse temperature $\beta$. Based on previous observations (Story et al., 2013) we also tested a variant in which dread itself is not discounted in time (i.e. omitting multiplication by $e^{-rt}$ in Equation S5, and thereby the first integral of Equation S6).

We also tested a variant of Loewenstein’s model based on hyperbolic discounting, which we term ‘Hyperbolic Dread’. This model makes the simplifying assumption that painful stimuli are instantaneous, not unreasonable given the brevity of painful outcomes in relation to the delays. The second is that dread itself is not discounted in time. Under this model, dread is given by the forward-looking integral of hyperbolically discounted expectation of pain. Instantaneous disutility from anticipation, is given by the expectation of pain at each moment, $t\leq d$:

$$A\left( x,d,t \right)=\frac{u\left( x \right)}{1+\rho(d-t)}$$

Dread is then given by the prospective integral of this:

$$D\left( x,d,L \right)=u\left( x \right)\frac{\alpha}{\rho}\log\left( 1+\rho d \right)$$

(S8)

Giving:

$$U\left( x,d \right)=u(x)\left[ \frac{1}{1+Kd}+ \frac{\alpha}{\rho}\log\left( 1+\rho d \right) \right]$$

(S9)

We note that this is equivalent to dread growing directly proportional to delay, under an assumption of logarithmic time perception^.^. The first term in the square bracket denotes hyperbolic discounting of pain with rate, $K$. The second first term represents the contribution of dread, where $\rho$ is a parameter governing the rate of accumulation of instantaneous anticipation over time, and $\alpha$ governs the overall contribution of dread. All the above parameters were bounded to be positive by exponential transformation.

We also tested a ‘Non-Scaled’ version of this model where the cost of waiting for pain scales with increasing delay, but not with increasing pain severity:

$$U\left( x,d \right)=\frac{\alpha}{\rho}\log\left( 1+\rho d \right)+ \frac{u(x)}{1+Kd}$$

(S10)

We note that in the above model the intensity of pain still influences its overall disutility, through the $u(x)$ term, though this does not modulate the specific disvalue derived from its anticipation.

**Bayesian Model Fitting Routine**

To fit alternative models of temporal and social discounting we used a hierarchical model-fitting approach, whereby the group-level distribution of each parameter is used as an empirical prior for individual-level parameter estimation. This approach allows the population -level distribution of data to constrain unreliable parameter estimates at the individual level. For each model, the probability of choosing an option, $i$, over the alternative option, $j$, depended on the relative utilities of the two options (derived from fitting the model) according to a standard softmax model of action selection:

$$\pi\left( choose i \right)= \frac{1}{1+exp[ \beta(U_{j}-U_{i})]}$$

(S11)

The degree of stochasticity in choices is governed by the inverse temperature parameter, $\beta$. At higher values of $\beta$ the decision-maker is more likely to select the option with higher utility; when $\beta$=0 the decision-maker is equally likely to choose either option, irrespective of their utilities, i.e. chooses randomly. $\beta$ can also be viewed as the weighting factor in a logistic regression, which maps from a continuous independent variable (utility difference) to a discrete dependent variable (choosing either option). The model fitting routine follows that previously described by Huys and colleagues (Huys et al., 2012), and is reproduced here. Each model yielded a parameter vector, $\theta_{i}$, for each subject, $i$. Parameters were constrained to be positive by exponential transform (inference and fitting was performed on the log of the parameters). In selected cases, where parameters were bounded to lie between 0 and 1, this was achieved by sigmoid transformation. Model fitting at the individual level aimed to find the maximum *a posteriori* estimate of $\theta_{i}$, given a vector of each subject’s choices, $C_{i}$:

$$\theta_{i}={argmax}_{\theta} p(C_{i}|\theta_{i})p(\theta_{i}|\vartheta)$$

(S12)

where $\vartheta$ are the parameters (hyperparameters) of an empirical normal prior distribution on $\theta$. We proceeded by estimating the maximum-likelihood hyperparameters, given the data from all$N$ subjects:

$\hat{\vartheta}^{ML}= {argmax}_{\vartheta} p\left( C_{1}\ldots C_{N} | \vartheta\right)= {argmax}_{\vartheta}\prod_{i} p(C_{i}|\vartheta)$

S(13)

where,

$$p\left( C_{i} | \vartheta\right)= \int d\theta_{i} p\left( C_{i} | \theta_{i} \right)p(\theta_{i}|\vartheta)$$

(S14)

The intractable integral above was estimated by Expectation-Maximization (E-M). The E-step at the $k$th iteration sought the maximum *a posteriori* (MAP) parameter estimates for each subject, given an estimate of the empirical priorfrom the preceding iteration, $\vartheta^{(k-1)}$:

$${\theta_{i}}^{(k)}= {argmax}_{\theta} p(C_{i}|\theta_{i})p(\theta_{i}|\vartheta^{(k-1)})$$

(S15)

This maximization was achieved by minimizing the negative log posterior probabilities by unconstrained nonlinear optimization in Matlab (Mathworks, MA, USA). To estimate the variance on the individual-level MAP parameters, we used a Laplace approximation, which assumes that the posterior is Gaussian around the MAP estimate:

$$p\left( \theta_{i} | C_{i} \right)\approx N\left( {\theta_{i}}^{\left( k \right)},\sum_{i}^{(k)} \right)$$

(S16)

where $\sum_{i}^{(k)}$ is the second moment around ${\theta_{i}}^{\left( k \right)},$ which approximates the variance. In the M-step, the estimated hyperparameters $\vartheta^{(k)}$ of the empirical prior distribution, mean $\mu,$and factorized variance, $\sigma^{2}$, were updated as follows:

$$\mu^{(k)}= \frac{1}{N}\sum_{i} {\theta_{i}}^{\left( k \right)}$$

(S15)

$$\left( \sigma^{(k)} \right)^{2}=\frac{1}{N}\sum_{i} \left[ \left( {\theta_{i}}^{\left( k \right)} \right)^{2}+\sum_{i}^{(k)} \right]-\left( \mu^{\left( k \right)} \right)^{2}$$

(S17)

We compared models by Bayesian model evidence, $p(C_{1}\ldots C_{N}|M)$, approximated as ${BIC}_{int}$:

$$-\frac{1}{2}{BIC}_{int}=\log p(C_{1}\ldots C_{N}\left| \hat{\vartheta}^{ML} \right)- \frac{1}{2}\left| M \right|log(\left| C_{1}\ldots C_{N} \right|)$$

(S18)

where $\left| C_{1}\ldots C_{N} \right|$ is the total number of choices made by all subjects, and $\left| M \right|$ is number of hyperparameters fitted. Notably here, by distinction from conventional BIC, $\log p(C_{1}\ldots C_{N}\left| \hat{\vartheta}^{ML} \right)$ is a sum over the model evidence at the subject level by integrating over subject-level parameters:

$$\log p(C_{1}\ldots C_{N}\left| \hat{\vartheta}^{ML} \right)= \sum_{i} \log\int d\theta p\left( C_{i} | \theta\right) p\left( \theta| \hat{\vartheta}^{ML} \right) \approx\sum_{i} \log\frac{1}{K}\sum_{k=1}^{K} p\left( C_{i} | \theta^{k} \right)$$

(S19)

The right hand expression approximates the integral by summing over $K$ samples, drawn from the empirical prior, $p\left( \theta| \hat{\vartheta}^{ML} \right)$. Thus the individual-level parameters intervene between the data and the group-level inference, but are averaged out when comparing models.

**Supporting Figures**


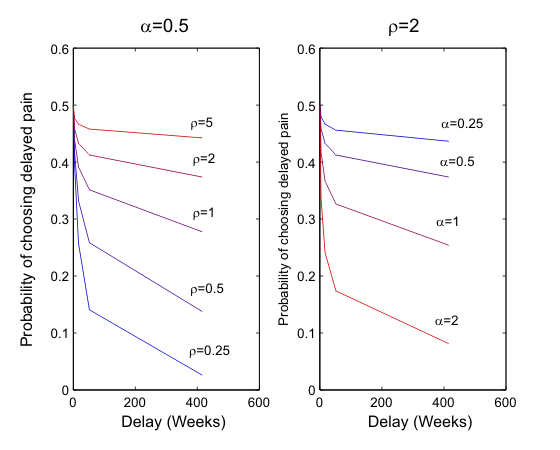


**Figure S1 Choice of delayed pain at varying parameter settings of the Non-scaled Hyperbolic Dread Model**

**Supporting References**

Huys, Q. J. *et al.* Bonsai trees in your head: how the Pavlovian system sculpts goal-directed choices by pruning decision trees. *PLoS Comput. Biol.*  **8**, e1002410 (2012).

Loewenstein, G. Anticipation and the valuation of delayed consumption. *Econ. J.* **97**, 666-684 (1987).

Story, G. W. *et al.* Dread and the Disvalue of Future Pain. *PLoS Comput. Biol.* **9**, e1003335 (2013).
